# Subretinal fluid disturbs the retinal venous blood flow in central serous chorioretinopathy

**DOI:** 10.1038/s41598-022-08865-y

**Published:** 2022-03-22

**Authors:** Joon Seo Lim, Cheolwon Moon, Junyeop Lee

**Affiliations:** 1 Clinical Research Center, Asan Medical Center, University of Ulsan College of Medicine, Seoul, South Korea; 2 Department of Ophthalmology, Yeungnam University Medical Center, Daegu, South Korea; 3 Department of Ophthalmology, Asan Medical Center, University of Ulsan College of Medicine, 88, Olympic-ro 43-gil, Songpa-gu, Seoul, 05505 South Korea; 4 Translational Biomedical Research Group, Biomedical Research Center, Asan Institute for Life Science, Asan Medical Center, Seoul, South Korea

**Keywords:** Macular degeneration, Retinal diseases

## Abstract

The significance of subretinal fluid in the retinal blood flow is unclear. Here, we evaluated the association between subretinal fluid (SRF) and retinal blood flow in eyes with central serous chorioretinopathy (CSC) using a retinal functional imager (RFI) and optical coherence tomography angiography (OCTA). In this retrospective case–control study involving 26 eyes from 18 CSC patients and 25 eyes from 21 age- and sex-matched controls, we found that the CSC group showed significant differences from the control group in terms of the retinal venule blood flow velocity (3.60 ± 0.43 vs 3.96 ± 0.56 mm/s; *p* = 0.030), retinal venule blood flow rate (8.75 ± 2.67 vs 12.51 ± 7.12 nl/s; *p* = 0.040), and the diameter of retinal venules (118.26 ± 14.25 vs 126.92 ± 35.31 μm; *p* = 0.045). Linear regression analysis showed that SRF thickness accounted for a 36.9% reduction in venous BFR (*p* = 0.013). The difference in the O_2_ saturation between retinal arteries and veins was greater in the CSC group. There was no correlation between SRF thickness and capillary densities in OCTA. Our findings suggest that disturbance in venous return and the associated altered oxygen may be significant changes in the retinal blood flow dynamics in eyes with SRF.

## Introduction

The healthy retina is maintained in a dehydrated and transparent state for optimal light transmission^1^. Fluid accumulation derived from an imbalance between the processes governing fluid entry and exit disrupts retinal neuronal integrity^1^. However, subretinal fluid (SRF) was associated with better visual outcomes in age-related macular degeneration (AMD)^2^. Although the presence of SRF was reported to reduce the risk of retinal atrophy in AMD^3^, this remains a controversial issue. Yet, there has been limited research that evaluated how the SRF affects retinal blood flow, which is essential for retinal homeostasis.

Central serous chorioretinopathy (CSC) is a condition belonging to the pachychoroid disease spectrum, which is characterized by thick choroid and associated retinal pathologies^4,5^. Subretinal fluid of CSC is caused by the permeable choriocapillaris, accompanied by retinal pigment epithelium (RPE) decompensation^6^. The natural course of CSC is often self-limiting and complete fluid reabsorption often occurs^7^. The visual acuity returns to normal within a few months following the resolution of fluid^8^. However, visual impairments may persist in some patients even after the dissipation of fluid, due to disruption of the retinal microarchitecture, subretinal fibrosis, and scarring or atrophy of the RPE^8–10^. There is still controversy among several studies using coherence tomography angiography (OCTA) whether the macular vascular alteration occurs at the early stage of CSC^9,11–14^. Thus, more sensitive and accurate imaging modalities are required for the early detection of changes in retinal blood flow.

Retinal functional imager (RFI) is a specialized imaging technique that allows non-invasive and direct measurement of retinal blood flow velocity (BFV), and is thus more advantageous than fluorescein angiography (FA) and OCTA^15^. RFI can avoid the risk of anaphylaxis resulting from the use of fluorescein dye^16^. In the multispectral imaging mode, RFI can also detect the differences between the absorption spectra of oxyhemoglobin and deoxyhemoglobin in order to determine the blood oxygenation status^16^. Therefore, RFI has clinical advantages over previous imaging techniques including OCTA and FA for investigating the retinal vascular pathophysiology.

Although recent studies reported that BFV is decreased in CSC-affected eyes, the changes in the BFV in each region and their relationship with other retinal vascular characters such as retinal capillary density and FAZ area are unknown^5^. Therefore, in this study, we evaluated the association between SRF and retinal blood flow in eyes with CSC. Specifically, we used RFI to investigate the changes in blood flow rate (BFR), BFV, and O_2_ saturation in CSC and their relationship with other retinal vascular characters.

## Results

### Baseline characteristics

Twenty-six eyes from 18 patients with CSC and 25 eyes from 21 age-matched controls were included in this study. Among the 26 eyes, 24 (92%) were chronic CSC (duration > 3 months). There were no significant differences between the two groups in terms of age, sex, refractory error, baseline BCVA, status of cataract surgery, hypertension, cardiovascular disease (Table 1). There were no cases of diabetes and cerebrovascular diseases in both groups. Baseline CMT was greater in the CSC group than in the control group (327.08 ± 79.51 μm vs 284.57 ± 36.37 mm/s, *p* = 0.027) (Table 1).

**Table 1:** Baseline demographics of the CSC patients and control group.

	Control (n = 25)	CSC (n = 26)	*P* value
Male sex, n (%)	21	24	0.32*
Age, years	52.9 ± 9.5	53.0 ± 11.9	0.90
Refractory error, diopter	−0.14 ± 1.47	0.10 ± 1.03	0.48
Baseline BCVA, logMAR	0.08 ± 0.21	0.15 ± 0.24	0.07
Basline IOP	15.32 ± 3.28	15.31 ± 2.84	0.66
Phakic:pseudophakic	25:0	22:4	0.06*
Diabetes	25:0	26:0	
Hypertension	20:5	20:6	0.53*
Cardiovascular disease	23:2	22:4	0.35*
Cerebrovascular disease	25:0	26:0	
Baseline CMT, μm	284.57 ± 36.37	327.08 ± 79.51	0.027
SRF thickness, μm	–	196.88 ± 75.18	
**Arterial BFR, nl/s**	−13.25 ± 5.60	−10.43 ± 3.08	0.07
Fovea	−11.43 ± 4.62	−9.31 ± 4.53	0.10
Parafovea	−15.72 ± 12.17	−12.74 ± 4.93	0.61
**Venous BFR, nl/s**	12.51 ± 7.12	8.75 ± 2.67	0.040
Fovea	10.68 ± 6.05	7.26 ± 3.26	0.023
Parafovea	14.56 ± 9.57	12.45 ± 9.25	0.31
**Arterial BFV, mm/s**	−4.51 ± 0.51	−4.39 ± 0.48	0.33
BFV-SA	−1.23 ± 0.56	−1.63 ± 0.68	0.49
BFV-MA	−2.79 ± 0.71	−2.80 ± 0.62	0.70
BFV-LA	−4.88 ± 0.38	−4.78 ± 0.50	0.44
Fovea	−4.59 ± 0.59	−4.33 ± 0.76	0.12
Parafovea	−4.46 ± 0.65	−4.63 ± 0.55	0.48
**Venous BFV, mm/s**	3.96 ± 0.56	3.60 ± 0.43	0.030
BFV-SV	1.35 ± 0.47	1.37 ± 0.64	0.86
BFV-MV	2.53 ± 0.62	2.42 ± 0.73	0.71
BFV-LV	4.19 ± 0.45	3.92 ± 0.42	0.045
Fovea	3.97 ± 0.68	3.53 ± 0.67	0.012
Parafovea	4.01 ± 0.61	3.85 ± 0.80	0.41
Arterial vessel diameter, μm	122.52 ± 17.94	114.59 ± 10.11	0.09
Venous vessel diameter, μm	126.92 ± 35.31	118.26 ± 14.25	0.045
**OCTA measurements**			
FCP density, %	54.40 ± 3.53	53.53 ± 3.45	0.41
SCP density, %	46.63 ± 3.95	47.03 ± 3.59	0.75
DCP density, %	48.87 ± 3.28	46.82 ± 4.70	0.25
FAZ area, mm^2^	0.29 ± 0.12	0.33 ± 0.13	0.15

*Pearson’s Chi-squared test. All other *P* values were calculated using Mann–Whitney test.

*BCVA* best-corrected visual acuity, *BFR* blood flow rate, *BFV* blood flow velocity, *BFV-SA* small arteriole blood flow velocity (diameter smaller than 40 μm), *BFV-MA* middle arteriole blood flow velocity (diameter between 40 and 80 μm), *BFV-LA* large arteriole blood flow velocity (diameter greater than 80 μm), *BFV-SV* small venule blood flow velocity (diameter smaller than 40 μm), *BFV-MV* middle venule blood flow velocity (diameter between 40 and 80 μm), *BFV-LV* large venule blood flow velocity (diameter greater than 80 μm), *CMT* central macular thickness, *CSC* central serous chorioretinopathy, *DCP* deep capillary plexus, *FAZ* foveal avascular zone, *FCP* full capillary plexus, *OCTA* optical coherence tomography angiography, *SCP* superficial capillary flexus, *SRF* subretinal fluid.

### Retinal blood vessels measured using RFI according to the presence of SRF

The CSC group and the control group did not show significant differences in the arterial BFV (−4.51 ± 0.51 mm/s vs −4.39 ± 0.48 mm/s, *p* = 0.33), BFR (−13.25 ± 5.60 nl/s vs −10.43 ± 3.08 nl/s, *p* = 0.07), and vessel diameter (122.52 ± 17.94 μm vs 114.59 ± 10.11 μm, *p* = 0.09) (Table 1). In the analysis according to vessel diameter size and region, the CSC group and the control group did not show significant differences in BFV (−1.63 ± 0.68 mm/s vs −1.23 ± 0.56 mm/s, *p* = 0.49 for BFV-SA; −2.80 ± 0.62 mm/s vs −2.79 ± 0.71 mm/s, *p* = 0.70 for BFV-MA; −4.78 ± 0.50 mm/s vs −4.88 ± 0.38 mm/s, *p* = 0.44 for BFV-LA; −4.33 ± 0.76 mm/s vs −4.59 ± 0.59 mm/s, *p* = 0.12 for arterial BFV of the fovea; −4.63 ± 0.55 mm/s vs −4.46 ± 0.65 mm/s, *p* = 0.48 for arterial BFV of the parafovea) and BFR (−9.31 ± 4.53 nl/s vs −11.43 ± 4.62 nl/s, *p* = 0.10 for arterial BFR of the fovea; −12.74 ± 4.93 nl/s vs −15.72 ± 12.17 nl/s, *p* = 0.61 for Arterial BFR of parafovea) (Table 1).

Venous BFV, BFR, and vessel diameter were smaller in the CSC group than in the control group (3.60 ± 0.43 mm/s vs 3.96 ± 0.56 mm/s, *p* = 0.030 for venous BFV; 8.75 ± 2.67 nl/s vs 12.51 ± 7.12 nl/s, *p* = 0.040 for venous BFR; 118.26 ± 14.25 μm vs 126.92 ± 35.31 μm, *p* = 0.045 for vessel diameter) (Table 1). In the analysis according to vessel diameter size, BFV-LV was smaller in the CSC group (3.92 ± 0.42 mm/s vs 4.19 ± 0.45 mm/s, *p* = 0.045) while BFV-SV and BFV-MV did not show significant differences between the two groups (1.37 ± 0.64 mm/s vs 1.36 ± 0.47 mm/s, *p* = 0.86 for BFV-SV; 2.42 ± 0.73 mm/s vs 2.53 ± 0.62 mm/s, *p* = 0.71 for BFV-MV) (Table 1). In the analysis according to region, foveal venous BFV (3.53 ± 0.67 mm/s vs 3.97 ± 0.68 mm/s, *p* = 0.012) and BFR (7.26 ± 3.26 nl/s vs 10.68 ± 6.05 nl/s, *p* = 0.023) were significantly smaller in the CSC group, while parafoveal venous BFV and BFR did not show significant differences between the two groups (3.85 ± 0.80 mm/s vs 4.01 ± 0.61 mm/s, *p* = 0.41 for venous BFV of the parafovea;12.45 ± 9.25 nl/s vs 14.56 ± 9.57 nl/s, *p* = 0.31 for venous BFR of the parafovea) (Table 1).

### *En face* angiographic features measured using OCTA according to the presence of SRF

The CSC group and the control group did not show significant differences in terms of the *en face* angiographic features measured using OCTA such as FCP density (74.35 ± 99.82% vs 54.40 ± 3.53%, *p* = 0.59), SCP density (47.03 ± 3.59% vs 46.63 ± 3.95%, *p* = 0.75), DCP density (46.82 ± 4.70% vs 48.87 ± 3.28%, *p* = 0.25), and FAZ area (0.33 ± 0.13 mm^2^ vs 0.29 ± 0.12 mm^2^, *p* = 0.15) (Table 1).

### Retinal vascular blood flow velocity and its associated factors

Linear correlation analysis did not reveal significant associations between arterial and venous BFV with age, sex, SRF thickness, SCP density, and FAZ area. However, arterial BFR and venous BFR were positively associated with arterial BFV and venous BFV, respectively. Arterial BFR showed a significant correlation with mean arterial BFV (R = 0.729, *p* < 0.001); when analysed according to vessel diameter size, arterial BFR showed a significant correlation with BFV-LA (R = 0.600, *p* < 0.001), but not with BFV-SA (R = −0.354, *p* = 0.20) and BFV-MA (R = −0.055, *p* = 0.75) (Table 2). Venous BFR showed a significant correlation with mean venous BFV (R = 0.734, *p* < 0.001); when analysed according to vessel diameter size, venous BFR showed a significant correlation with BFV-LV (R = 0.75, *p* < 0.001), but not with BFV-SV (R = −0.041, *p* = 0.87) and BFV-MV (R = −0.017, *p* = 0.92) (Table 2).

**Table 2:** Arterial blood flow velocity and its associated factors.

	Mean arterial BFV		BFV-SA		BFV-MA		BFV-LA	
	R	*P* value	R	*P* value	R	*P* value	R	*P* value
Age	−0.287	0.06	0.270	0.33	0.291	0.09	−0.193	0.21
Sex	−0.193	0.22	−0.112	0.69	−0.125	0.47	−0.024	0.88
CMT	−0.210	0.19	−0.259	0.35	0.351	0.039	−0.111	0.48
SRF thickness	−0.350	0.18	−0.789	0.11	0.367	0.22	−0.428	0.10
Arterial BFR	0.729	< 0.001	−0.354	0.20	−0.055	0.75	0.600	< 0.001
Arterial vessel diameter	−0.701	< 0.001	0.378	0.17	0.164	0.34	−0.521	< 0.001
**OCTA measurements**								
FCP density (%)	−0.034	0.84	−0.431	0.14	0.547	0.001	−0.221	0.18
SCP density (%)	0.200	0.24	−0.188	0.54	−0.264	0.15	0.188	0.25
DCP density (%)	0.023	0.89	−0.223	0.46	−0.065	0.72	0.004	0.98
FAZ area (mm^2^)	−0.097	0.57	0.214	0.48	−0.243	0.18	−0.024	0.88

All P values were calculated using Pearson's correlation test.

*BFR* blood flow rate, *BFV* blood flow velocity, *BFV-SA* small arteriole blood flow velocity (diameter smaller than 40 μm), *BFV-MA* middle arteriole blood flow velocity (diameter between 40 and 80 μm), *BFV-LA* large arteriole blood flow velocity (diameter greater than 80 μm), *CMT* central macular thickness, *CSC* central serous chorioretinopathy, *DCP* deep capillary plexus, *FAZ* foveal avascular zone, *FCP* full capillary plexus, *OCTA* optical coherence tomography angiography, *SCP* superficial capillary flexus, *SRF* subretinal fluid.

Arterial vessel diameter showed a significant negative correlation with mean arterial BFV (R = −0.701, *p* < 0.001) and BFV-LA (R = −0.521, *p* < 0.001), but not with BFV-SA (R = 0.378, *p* = 0.17) and BFV-MA (R = 0.164, *p* = 0.34). Venous vessel diameter showed a significant positive correlation with mean venous BFV (R = 0.598, *p* < 0.001) and BFV-LV (R = 0.542, *p* < 0.001), but not with BFV-SV (R = −0.097, *p* = 0.70) and BFV-MV (R = 0.309, *p* = 0.07) (Table 2).

FCP density showed a positive correlation with BFV-MA (R = 0.547, *p* = 0.001; Table 2) and a negative correlation with mean venous BFV (R = −0.473, *p* = 0.003; Table 3). DCP density was significantly correlated with mean venous BFV (R = 0.332, *p* = 0.045) (Table 3).

**Table 3:** Venous blood flow velocity and its associated factors.

	Mean venous BFV		BFV-SV		BFV-MV		BFV-LV	
	R	*P* value	R	*P* value	R	*P* value	R	*P* value
Age	0.135	0.39	0.189	0.45	−0.302	0.07	0.170	0.27
Sex	0.122	0.43	–	–	−0.029	0.87	−0.064	0.68
CMT	−0.133	0.41	0.110	0.67	−0.031	0.86	−0.091	0.57
SRF thickness	−0.427	0.10	0.013	0.97	−0.067	0.81	−0.426	0.10
Venous BFR	0.734	< 0.001	−0.041	0.87	−0.017	0.92	0.748	< 0.001
Venous vessel diameter	0.598	< 0.001	−0.097	0.70	0.309	0.07	0.542	< 0.001
**OCTA measurements**								
FCP density (%)	−0.473	0.003	−0.018	0.95	−0.232	0.18	−0.251	0.13
SCP density (%)	0.195	0.25	−0.180	0.51	0.234	0.18	0.107	0.52
DCP density (%)	0.332	0.045	0.152	0.57	0.267	0.12	0.201	0.23
FAZ area (mm^2^)	−0.086	0.61	0.208	0.44	−0.068	0.70	−0.235	0.16

All P values were calculated using Pearson's correlation test.

*BFR* blood flow rate, *BFV* blood flow velocity, *BFV-SV* small venule blood flow velocity (diameter smaller than 40 μm), *BFV-MV* middle venule blood flow velocity (diameter between 40 and 80 μm), *BFV-LV* large venule blood flow velocity (diameter greater than 80 μm), *CMT* central macular thickness, *CSC* central serous chorioretinopathy, *DCP* deep capillary plexus, *FAZ* foveal avascular zone, *FCP* full capillary plexus, *OCTA* optical coherence tomography angiography, *SCP* superficial capillary flexus, *SRF* subretinal fluid.

### Relationship between SRF thickness and retinal BFV and BFR

**Figure 1:**
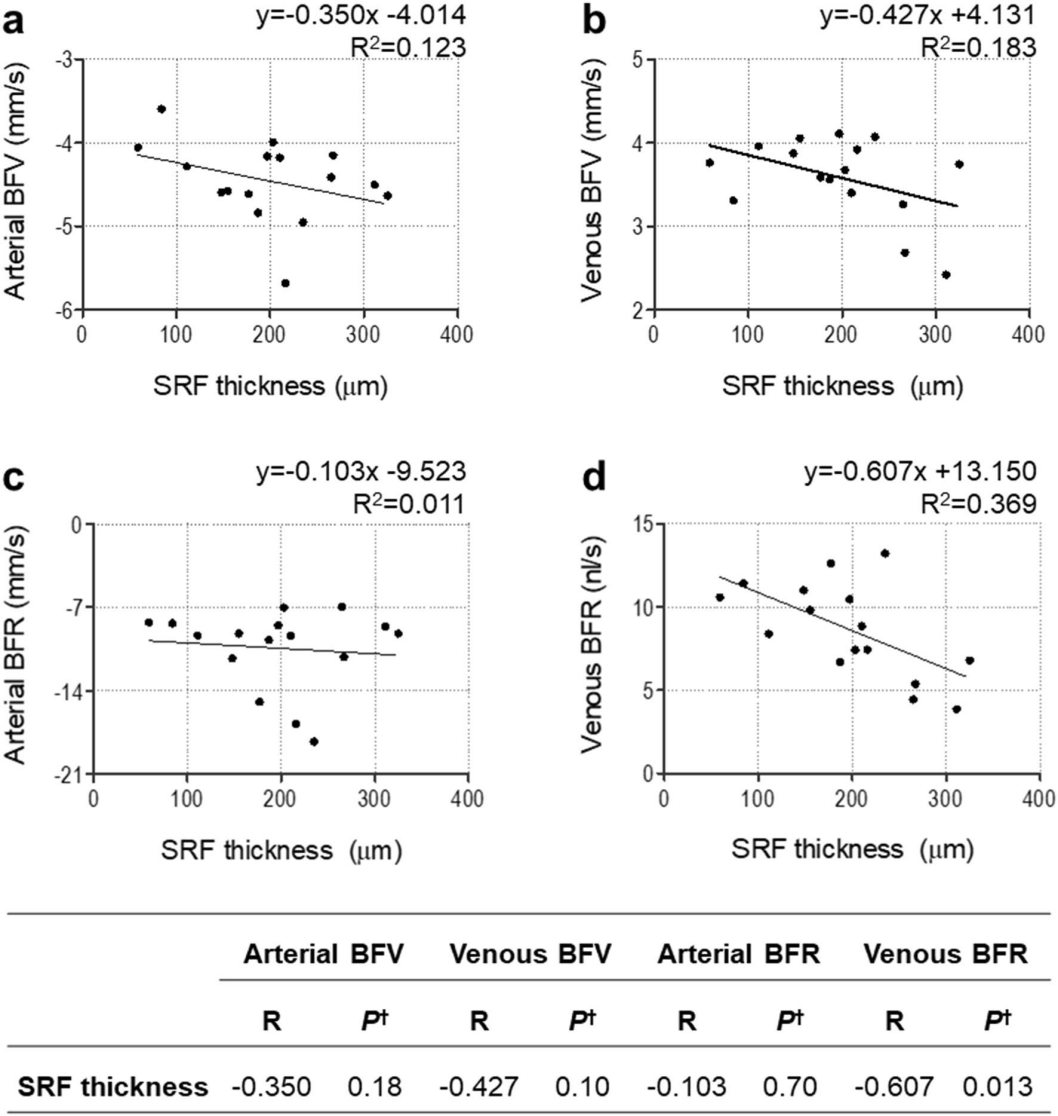
Relationship between subretinal fluid (SRF) thickness and arterial blood flow velocity **(a)**, venous blood flow velocity **(b)**, arterial blood flow rate **(c)**, and venous blood flow rate **(d)** in central serous chorioretinopathy. ^†^Pearson’s correlation. *BFR* blood flow rate, *BFV* blood flow velocity, *SRF* subretinal fluid.

Simple linear regression analysis showed a significant negative correlation between SRF thickness and venous BFR (R = −0.607, R^2^ = 0.369, *p* = 0.013; Fig. 1d). In contrast, SRF thickness showed weak correlations with arterial BFV (R = −0.350, R^2^ = 0.123, *p* = 0.18), arterial BFR (R = −0.103, R^2^ = 0.011, *p* = 0.70), and venous BFV (R = −0.427, R^2^ = 0.183, *p* = 0.10; Fig. 1a–c).

### Relationship between SRF thickness and OCTA parameters

**Figure 2:**
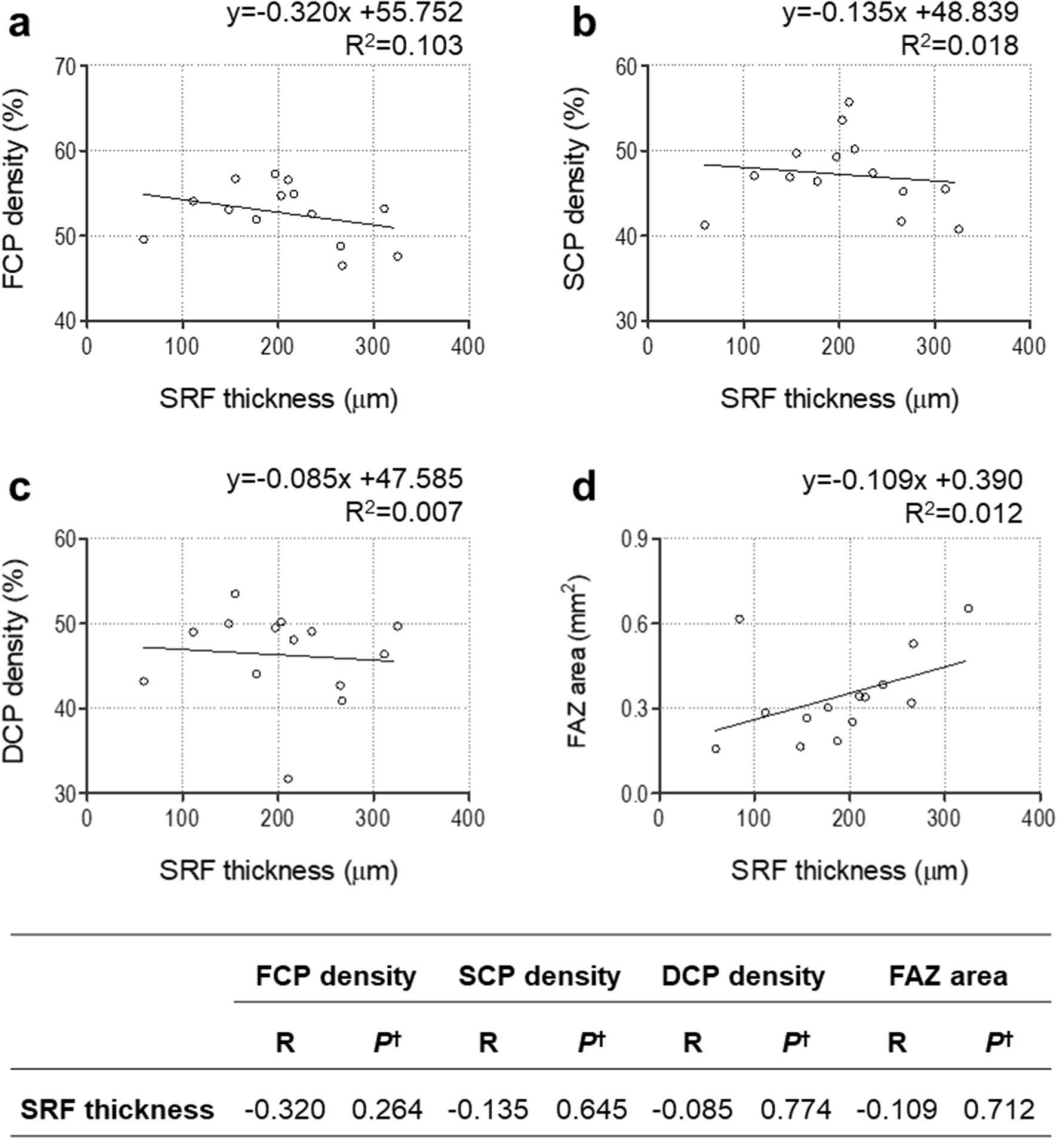
Relationship between subretinal fluid (SRF) thickness and OCTA parameters: FCP density **(a)**, SCP density **(b)**, DCP density **(c)**, and FAZ area **(d)** in central serous chorioretinopathy. ^†^Pearson’s correlation. *SRF* subretinal fluid, *DCP* deep capillary plexus, *FAZ* foveal avascular zone, *FCP* full capillary plexus, *SCP* superficial capillary flexus, *SRF* subretinal fluid.

Retinal capillary densities (FCP, SCP, and DCP) decreased as the SRF thickness increased, albeit without a statistically significant correlation (Fig. 2a–c). The FAZ area was also not significantly associated with the SRF thickness (Fig. 2d).

### Representative cases

#### Case 1

**Figure 3:**
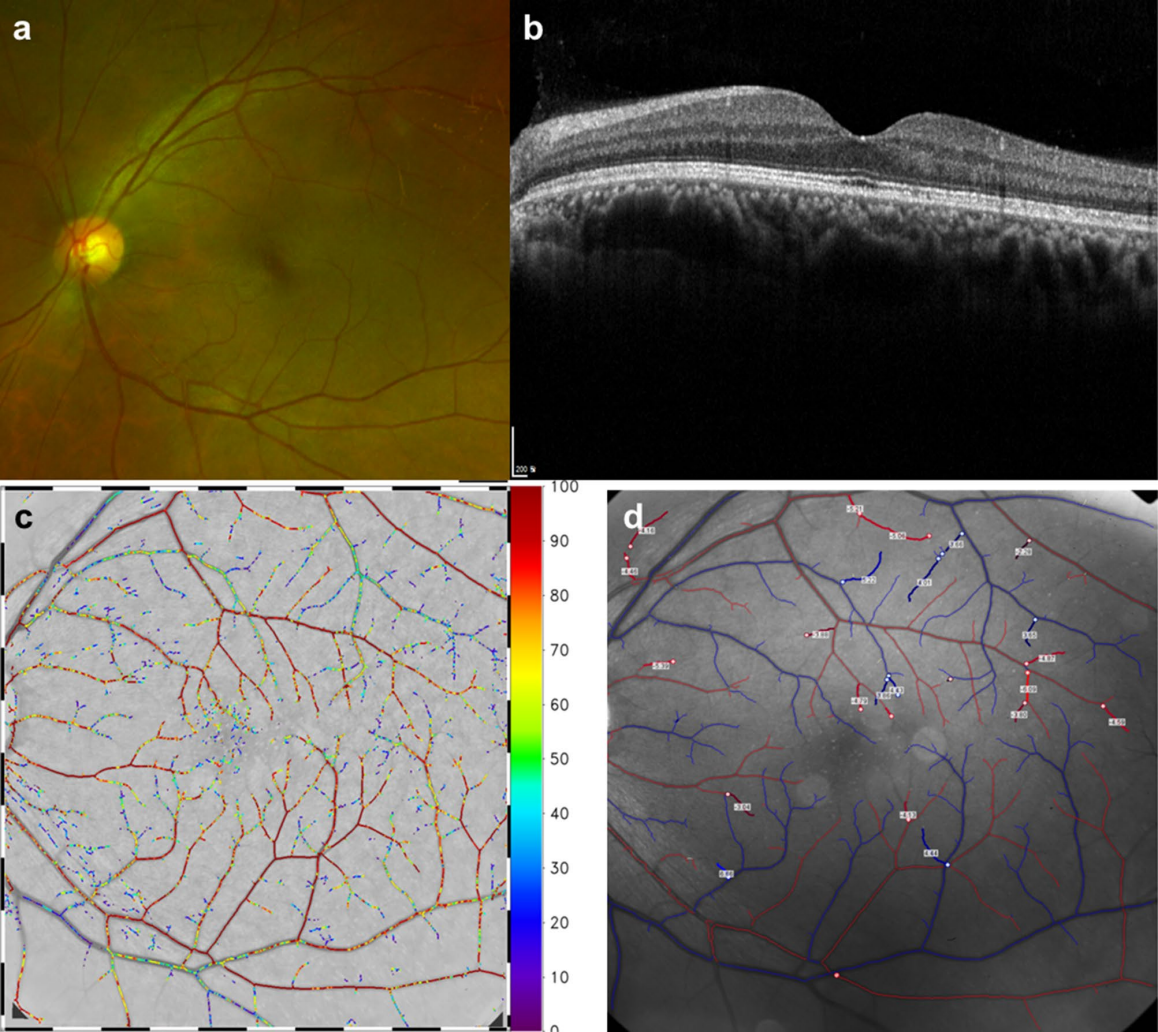
Retinal angiographic images from age-matched control group using fundus photography **(a)**, optical coherence tomography **(b)**, and retinal functional imager (RFI) **(c,d)**. **(c)** O_2_ saturation image and **(d)** retinal vessel velocity image.

A 47-year-old male presented with blurred vision on his right eye for 6 months. On examination, the visual acuity was 20/20 in the left eye. The anterior segments of both eyes were found to be normal by slit-lamp biomicroscopy. Fundus examination of the left eye was normal (Fig. 3a). OCT revealed normal in the left eye (Fig. 3b). O_2_ saturation image was taken by RFI (Fig. 3c). In red are the arteries that do not lose oxygen. Small venules near the fovea still have high oxygen saturation because they are very close to the arterial capillary. Blood of small venules drained to the superior and inferior retinal arcade veins. The O_2_ saturation of superior and inferior retinal arcade veins reached 25–75%. The number of retinal vein segments of blue color was lesser than that of the retinal vein segments of green-to-red color (Fig. 3c). Arterial BFV of the left eye was −4.674 mm/s and venous BFV of the same eye was 4.501 mm/s (Fig. 3d).

#### Case 2

**Figure 4:**
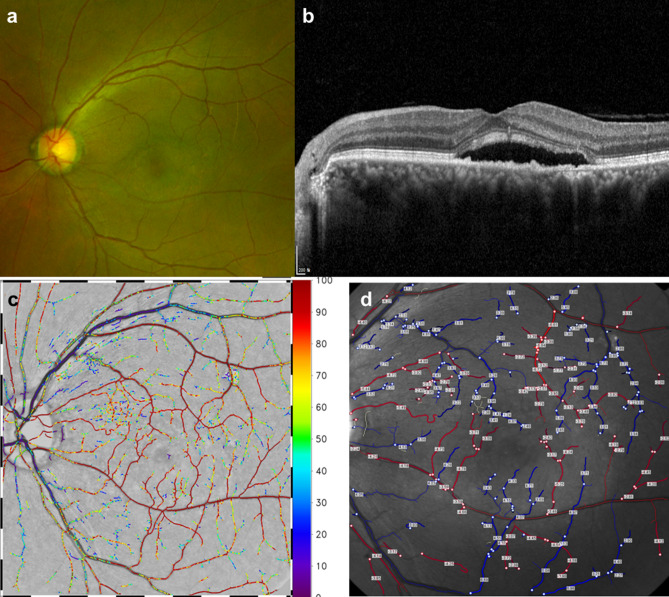
Retinal angiographic images from a case of central serous chorioretinopathy using fundus photography **(a)**, optical coherence tomography **(b)**, and retinal functional imager (RFI) **(c,d)**. **(c)** O_2_ saturation image and **(d)** retinal vessel velocity image.

A 50-year-old male presented with blurred vision on his left eye for 2 years. On examination, the visual acuity was 20/30 in the left eye. The anterior segments of both eyes were found to be normal by slit-lamp biomicroscopy. Fundus examination and OCT of the left eye showed well-demarcated SRF on macular (Fig. 4a,b). O_2_ saturation image was taken by RFI (Fig. 4c). The color of O_2_ saturation of retinal arteries was darker than that of Case 1. The O_2_ saturation of retinal arteries near the fovea was greater than that of Case 1 (Figs. 2c, 3c). Small venules near the fovea and crossing arteries still had high oxygen saturation (Fig. 4c). The O_2_ saturation of superior and inferior retinal arcade veins reached a similar level with that of Case 1, but the number of retinal vein segments of blue color was greater than that of the retinal vein segments of green-to-red color (Fig. 4c). Arterial BFV of the left eye was −3.991 mm/s and venous BFV of the same eye was 3.676 mm/s (Fig. 4d).

## Discussion

CSC is characterized by delayed choroidal infusion, choroidal vascular hyperpermeability, and choroidal venous dilation, which suggests that the choroid is primarily involved in the pathophysiology of CSC^20,21^. However, with the development of OCTA, recent studies have reported that CSC cases have blood flow abnormalities in the external retina^22^. As an example, Nelis et al. reported an increase in retinal flow density and a decrease in the FAZ in both the affected and unaffected eyes of CSC patients^9^. Therefore, retinal vascular problems may also be involved in the pathophysiology of CSC.

Several imaging techniques are available for retinal vascular analysis, including FA, OCTA, adaptive optics with scanning light ophthalmoscopy, and laser speckle flow cytometry. Compared with these imaging techniques, RFI has advantages in quantitative measurement of the blood flow velocity and oxygen saturation^23–26^. Moreover, RFI is non-invasive and does not require intravenous fluorescein dye injection^18,26^. However, RFI has a disadvantage compared with OCTA in terms of 3D imaging and volumetric imaging^17,27^. In the present study, we performed the measurement of BFV and BFR using RFI in eyes with or without CSC. As expected, venous BFR and BFV in the fovea were significantly smaller in the CSC group than in the control group. Arterial BFV was positively correlated with BFR and negatively correlated with arterial vessel diameter. Venous BFV was positively correlated with BFR and venous vessel diameter. DCP density was positively correlated with venous BFV, and SRF thickness was negatively correlated with venous BFR.

Beutelspacher et al. observed that the retinal BFV in the retinal veins was significantly smaller in CSC, especially in the larger retinal veins, while retinal arterial BFV was not significantly different between CSC patients and controls^5^. The result of the present study is in line with the previous study as we found that the diameters of retinal veins were smaller in CSC, while those of retinal arteries were not significantly different from the control group (Table 1). We assume that the lack of significant differences in BFV, BFR, and vessel diameter in retinal arteries in CSC is due to the presence of autoregulation in retinal arteries^28–30^. In contrast, the reason for the significant decreases in BFV, BFR, and vessel diameter of retinal veins in CSC is likely due to the mass effect of SRF or lack of autoregulation in the retinal veins. To investigate the mass effect of SRF on retinal arterial and venous BFV and BFR, we performed a linear regression analysis (Fig. 1) and found a correlation between SRF thickness and the BFR of retinal veins (*R*^2^ = 0.369). This result suggests that while the weight of SRF may decrease the BFR of retinal veins, the presence of autoregulation of retinal arteries may prevent the decrease in the BFR and BFV of retinal arteries. The selective decrease of venous BFV and BFR in the fovea of CSC patients may also be related to the mass effect of SRF (Table 1).

Previous studies using OCTA reported contrasting results on the change of DCP density in CSC. Piccolino et al. showed that DCP density was lower in CSC patients compared with healthy controls^14^, while Nelis et al. showed that CSC patients had higher DCP density and larger FAZ area than did healthy controls^9^. In our study, CSC patients had a lower FCP density, smaller FAZ area, and higher DCP density than did healthy controls, albeit without statistical significance (Table 1). The lack of significant difference in the DCP density and FAZ area according to the presence of CSC may be due to the small sample size. Nelis et al. suggested that the decrease in FAZ and increase in DCP density in CSC may be due to pathological changes in CSC such as accumulation of SRF^9^, and Eperon et al. suggested that continuous SRF may stimulate an increase in retinal flow density^31^. There was no correlation between SRF thickness and capillary densities in our study.

In our study, arterial BFV in CSC patients was negatively correlated with the arterial vessel diameter, especially in large retinal arteries, which may be the result of the autoregulation of retinal arteries. If the BFR of retinal arteries is increased, the autoregulation system may suppress the input of blood flow by decreasing the retinal arterial vessel diameter in order to maintain the BFR of retinal capillaries at a certain level. Also, venous BFV was positively correlated with venous BFR, venous vessel diameter, and DCP density. The DCP was organized into capillary vortexes (i.e., radial convergence of capillaries toward an epicenter), which drain into the superficial venules^32^. Therefore, as the vascular DCP increases, the BFR and BFV of retinal veins may increase because of the increased flow from the DCP.

The difference in the O_2_ saturation between retinal arteries and veins was greater in CSC patients than in controls. Li et al. used a non-invasive retinal oximeter (Oxymap T1, Oxymap ehf., Reykjavik, Iceland) to show that in the eyes of CSC patients, the O_2_ saturation of retinal arteries was increased in the inferotemporal quadrant and the O_2_ saturation of retinal veins was decreased in the inferonasal quadrant^33^. In addition, O_2_ saturation of retinal arteries also increased around the macular region, suggesting that these contribute to the pathophysiology of CSC^33^. Our study showed similar results with Li et al.’s study. Turkcu et al. found that the antioxidant capacity was significantly decreased in CSC cases, suggesting that the antioxidant defense system may be inadequate or corrupted in CSC^34^. Moreover, lower retinal vein velocity leads to more residence time of blood flow, which may further drive the difference in O_2_ saturation between retinal arteries and veins. It is also possible that the surrounding SRF may dilute the retinal venous O_2_ saturation.

Our study has several limitations. First, this study could have been underpowered to detect small differences according to the presence of CSC because of the small sample size. Most of the eyes had chronic CSC, and parameters such as blood pressure and heart rate were not measured in this study. As recruiting larger cohorts of CSC patients may be a challenge in single centers, multicenter studies on this issue are warranted. Nevertheless, this study provides the information of correlation between BFV, BFR, and the retinal capillary density and SRF thickness in CSC eyes. Second, RFI is non-invasive but relatively time-consuming (2–10 min) and thus requires patience in the examinees. Lastly, because CSC predominantly affects people aged between 20 and 50 years, there could have been a selection bias in the patient group; nevertheless, we tried to overcome this issue by matching the control group by age.

In conclusion, we found that SRF in eyes with CSC affected the retinal homeostasis through alterations in blood flow. Venous BFV, BFR, and vessel diameter were lower in eyes with CSC than healthy eyes. The SRF thickness was associated with reduced venous BFR, and CSC may aggravate the SRF due to venous stasis. Compared with OCTA, RFI had advantages in detecting subtle changes in retinal blood flow in the presence of SRF. The difference in O_2_ saturation between retinal arteries and veins was greater in CSC than in controls. These findings suggest that retinal venous problems and the associated altered oxygen metabolism may be significant changes in the retinal blood flow dynamics in eyes with SRF.

## Methods

### Subjects

This retrospective study included patients with CSC and age- and sex-matched controls. The data were collected between October 2019 and February 2020 at the Department of Ophthalmology in Yeungnam University Hospital (Daegu, South Korea). Of the patient data, we selected those from patients who had undergone OCT, OCTA, and RFI. All patients had undergone comprehensive ophthalmic examinations including best-corrected visual acuity (BCVA) measurement, dilated fundus examination, spectral-domain OCT (SD-OCT, Spectralis; Heidelberg Engineering, Heidelberg, Germany), OCTA (Optovue, Inc, Fremont CA, USA), and RFI 3005 (Optical Imaging Ltd., Rehovot, Israel). This retrospective study was approved by the institutional review board of Yeungnam University Hospital and adhered to the tenets of the Declaration of Helsinki for research involving human subjects. Informed consent was obtained from all subjects. All the methods were performed in accordance with relevant guidelines/regulations.

The clinical diagnosis of CSC was based on the presence of subretinal fluid (SRF) with or without pigment epithelium detachment (PED) accompanied by local and/or diffuse leakage in FFA and ICGA. The exclusion criteria were as follows: (1) retinal diseases that affect ocular circulation such as diabetic retinopathy and retinal vein occlusion, (2) concomitant retinal disease such as retinal detachment, macular hole, epiretinal membrane, and glaucoma, (3) refractory error more than 3.5 diopters, (4) massive subretinal hemorrhage or fibrosis obscuring the choroidal vasculature on RFI and OCTA, (5) severe media opacity that could degrade image quality, and (6) history of treatments that can cause significant changes to choroidal status such as intraocular surgery. Cataract surgery performed more than 3 months previously was not considered an exclusion criterion. Age-matched patients without ocular disease as confirmed by history and ophthalmic examinations who visited our clinic for a health-screening checkup were included as healthy controls. We randomly selected one of the two eyes in the healthy control group.

### Retinal microvascular blood flow and O_2_ saturation analysis using RFI

#### Flowmetry

The RFI system (Optical Imaging, Rehovot, Israel) is composed of a fundus camera, stroboscopic illumination, and a fast filter wheel^17^. Fast stroboscopic illumination enables the camera to take multiple snapshots of the retina in less than 0.2 s to track the movement of red blood cells through each sequential frame^16,18^. Using multiple sequences, RFI creates capillary perfusion maps and semi-automatically assesses blood flow velocity in arterioles and venules^16^. The instrument’s analysis software provides quantitative analysis of the BFV that includes the following: BFV in each segmental artery, vein, and total, identification number of each segment that was marked, and the diameter of each blood vessel.

To assess BFV in each vessel diameter, arteries and veins were subgrouped according to their diameter as follows: small arteriole BFV (BFV-SA, diameter smaller than 40 μm), middle arteriole BFV (BFV-MA, diameter between 40 and 80 μm), large arteriole BFV (BFV-LA, diameter greater than 80 μm), small venule BFV (BFV-SV, diameter smaller than 40 μm), middle venule BFV (BFV-MV, diameter between 40 and 80 μm), large venule BFV (BFV-LV, diameter greater than 80 μm).

To assess the regional BFV and BFR, a grid with a 1.5-mm diameter ring centered on the fovea was applied to define two zones—fovea (interior area of the 1.5-mm diameter ring) and parafovea (exterior area of the 1.5-mm diameter ring).

#### Oximetry

Qualitative blood oximetry maps were generated from different reflections of the retinal vasculature using varying wavelengths according to previously described protocols^16^.

### Central macular thickness and subretinal fluid thickness measurement

CMT and SRF thickness was measured in the subfoveal region using OCT. CMT was defined as the vertical distance between the internal limiting membrane and the top of the RPE at the fovea. CMT was quantitatively analyzed by a custom-built software program (Heidelberg Eye Explorer, Version 1.9.10, Heidelberg Engineering, Heidelberg, Germany). SRF thickness was defined as the greatest distance between the top of the SRF and the top of the RPE at the fovea. Measurement of SRF thickness was performed using the built-in caliper tool in the Heidelberg Eye Explorer program at a single point below the fovea by an independent masked grader (C.M.). The supervising grader (J.L.) confirmed the final decision.

### Measurement of vessel density and area of foveal avascular zone

OCTA (Optovue, Inc, Fremont, CA, USA) was performed using the split spectrum amplitude-decorrelation angiography algorithm. For each eye, 3 × 3 mm-sized images were taken and analyzed at the full capillary plexus (FCP) and two capillary layers—superficial capillary plexus (SCP) and deep capillary plexus (DCP). FCP was defined as the retinal layer between the internal limiting membrane and the RPE. SCP was imaged with an *en face* section starting at the inner border of the ganglion cell layer to the inner border of the inner plexiform layer in the macular area. *En face* images of the DCP were obtained by segmenting from the inner boundary of the inner plexiform layer to the outer boundary of the outer plexiform layer^19^.

The segmentation was identified automatically. Vascular density was calculated as the percentage of the area occupied by vessels in the total area of images and the selected depth of vessels. Vascular density was calculated in 3 × 3 mm-sized images in the SCP, DCP, and FCP. FAZ area was defined as the area of capillary-free region demarcated by a ring of interconnecting capillaries at the margin of the fovea. Measurement of the SCP density, DCP density, FCP density, and FAZ area was performed using *en face* imaging and EnView data analysis software (AngioAnalytics, Optovue).

### Statistical analysis

Statistical analyses were performed using IBM SPSS V.20.0 for Windows (IBM Co., Armonk, NY, USA). The Mann–Whitney test and Chi-squared test were used for comparison of numerical variables between CSC patients and age-matched healthy controls. Pearson’s correlation test was performed between numerical variables and BFV of each size of vessels. Linear regression analysis was performed between SRF thickness and arteriole and venule BFV and BFR. Variables with a *p* value < 0.05 were considered statistically significant. All results are presented as mean ± standard deviation.

## Data Availability

All data generated or analyzed during this study are included in this published article. Additional datasets or raw files during the current study are available from the corresponding author on reasonable request.
